# Early Life Ozone Exposure Results in Dysregulated Innate Immune Function and Altered microRNA Expression in Airway Epithelium

**DOI:** 10.1371/journal.pone.0090401

**Published:** 2014-03-04

**Authors:** Candice C. Clay, Kinjal Maniar-Hew, Joan E. Gerriets, Theodore T. Wang, Edward M. Postlethwait, Michael J. Evans, Justin H. Fontaine, Lisa A. Miller

**Affiliations:** 1 California National Primate Research Center, University of California Davis, Davis, California, United States of America; 2 Department of Anatomy, Physiology, and Cell Biology, School of Veterinary Medicine, University of California Davis, Davis, California, United States of America; 3 Department of Environmental Health Sciences, School of Public Health, University of Alabama, Birmingham, Alabama, United States of America; Cincinnati Children's Hospital Medical Center, United States of America

## Abstract

Exposure to ozone has been associated with increased incidence of respiratory morbidity in humans; however the mechanism(s) behind the enhancement of susceptibility are unclear. We have previously reported that exposure to episodic ozone during postnatal development results in an attenuated peripheral blood cytokine response to lipopolysaccharide (LPS) that persists with maturity. As the lung is closely interfaced with the external environment, we hypothesized that the conducting airway epithelium of neonates may also be a target of immunomodulation by ozone. To test this hypothesis, we evaluated primary airway epithelial cell cultures derived from juvenile rhesus macaque monkeys with a prior history of episodic postnatal ozone exposure. Innate immune function was measured by expression of the proinflammatory cytokines IL-6 and IL-8 in primary cultures established following *in vivo* LPS challenge or, in response to *in vitro* LPS treatment. Postnatal ozone exposure resulted in significantly attenuated IL-6 mRNA and protein expression in primary cultures from juvenile animals; IL-8 mRNA was also significantly reduced. The effect of antecedent ozone exposure was modulated by *in vivo* LPS challenge, as primary cultures exhibited enhanced cytokine expression upon secondary *in vitro* LPS treatment. Assessment of potential IL-6-targeting microRNAs miR-149, miR-202, and miR-410 showed differential expression in primary cultures based upon animal exposure history. Functional assays revealed that miR-149 is capable of binding to the IL-6 3′ UTR and decreasing IL-6 protein synthesis in airway epithelial cell lines. Cumulatively, our findings suggest that episodic ozone during early life contributes to the molecular programming of airway epithelium, such that memory from prior exposures is retained in the form of a dysregulated IL-6 and IL-8 response to LPS; differentially expressed microRNAs such as miR-149 may play a role in the persistent modulation of the epithelial innate immune response towards microbes in the mature lung.

## Introduction

Ozone is a common inhaled air pollutant that is known to negatively impact respiratory health and contribute towards increased mortality in humans [1], [2], [3]. Epidemiologic studies have demonstrated a clear association between episodes of high ozone exposure and increased hospitalizations for respiratory illnesses such as exacerbations of asthma [4], [5]. The interaction between ozone exposure and the pulmonary immune system is complex; both suppressive and stimulatory effects have been described [6], [7]. Most experimental evidence supports a role for ozone in the enhancement of susceptibility to respiratory infections [8], [9], with numerous rodent models exhibiting impaired pulmonary microbial clearance upon exposure [10], [11], [12]. The mechanisms by which ozone exposure compromises host defense in the lung are not well understood. Reduced bacterial clearance is primarily attributed to ozone-mediated impairment of macrophage phagocytosis [10], although modulation of adaptive immune responses following exposure in humans has also been reported [13], [14].

Young children, with their immature mucosal immune system and limited host defense capacity, may be more sensitive to the immunomodulatory effects of ozone exposure [15], [16]. In addition, several physiological parameters including differences in breathing patterns, ventilation rates and lung surface area per unit body weight can result in enhanced ozone exposure in children compared to adults [17]. The first year of life represents a highly dynamic phase for both the respiratory and mucosal immune systems. Many consider this a “window of susceptibility” for modulation by the environment [18], [19], as postnatal irritant or toxicant exposure has the potential to permanently affect the growth trajectory and function of the respiratory system [20], [21]. Given the challenges and ethical concerns involved in studying pediatric populations, our knowledge of infant pulmonary immunity is largely restricted to studies of neonatal laboratory animals. Because rodents and humans exhibit substantial differences in the postnatal maturation of both pulmonary and immune systems, it is critical to address the impact of environmental exposures on development of respiratory tract immunity in a primate species.

We have previously shown that ozone exposure of rhesus monkeys during the postnatal period of development results in altered immune cell composition in both peripheral blood and bronchoalveolar lavage, with significantly increased monocytes in both compartments that persisted with maturity [20]. Despite the higher monocyte frequency in animals with a history of ozone exposure, the peripheral blood and airway inflammatory response to inhaled lipopolysaccharide (LPS) was significantly attenuated. Furthermore, we demonstrated that the lasting effects of ozone were retained in the peripheral blood compartment, as *ex vivo* LPS treatment of peripheral blood mononuclear cells collected 6 months after ozone exposure showed significantly reduced proinflammatory cytokine responses. In this current study, we have focused on the long term impact of postnatal ozone exposure on the airway epithelium, using our previously described rhesus monkey model.

The epithelial cell of the conducting airways may play a significant role in the development of exposure-related effects, as it is architecturally and functionally poised to serve as a liaison between the external environment and the immune system. Acute inhalation of ozone has been shown to result in direct airway epithelial damage, with loss of ciliary function, enhanced permeability and impaired mucociliary clearance [7]. While the deleterious outcomes of ozone on airway epithelium have been documented, reports of ozone effects on epithelial cell immune responses are variable [22], [23], [24] and establishment of a persistent immunomodulatory phenotype in association with exposure has not been investigated. There is growing evidence to suggest that the respiratory tract develops a subtly unique inflammatory profile that is shaped by our prior exposures [25]. Although immunological memory is often attributed to the adaptive immune response, molecular programming of epithelial cell genes that likely impact innate immunity has been reported in asthmatics and cigarette smokers [26], [27]. Epigenetics and transcriptional regulation by microRNAs may, in part, explain the causal role of environmental exposures in alteration of epithelial cell phenotypes [28].

Given that the molecular profile of the airway epithelial cell may vary in association with chronic lung disease, we hypothesized that postnatal ozone exposure intrinsically alters the ability of airway epithelial cells to respond to a microbial challenge, and that this may enhance susceptibility to respiratory infection with maturity. In the present study, we focused on the cellular response to LPS, as this molecule is ubiquitous in the environment and is recognized by the pathogen pattern receptor Toll-like receptor 4 (TLR4). We tested our hypothesis using primary airway epithelial cell cultures derived from juvenile monkeys that were exposed to episodic ozone during the first six months of life, followed by filtered air housing until one year of age. Both *in vivo* and *in vitro* approaches were used to address the question of whether postnatal air pollutant exposures persistently alter the ability of airway epithelial cells to synthesize proinflammatory cytokines in response to exogenous LPS.

## Materials and Methods

### Ethics Statement

All animal procedures were approved by the University of California, Davis, Institutional Animal Care and Use Committee (Protocol #06-12245). Care and housing of animals before, during, and after treatment complied with the provisions of the Institute of Laboratory Animal Resources and conforms to practices established by the Association for Assessment and Accreditation of Laboratory Animal Care International (AAALAC International).

### Animal Exposure

Male rhesus macaque (*Macaca mulatta*) monkeys born at the California National Primate Research Center (CNPRC) were randomized into exposure groups based on birth order. Power projections at alpha level = 0.05 with POWERLIB202 software (University of North Carolina Chapel Hill) were used to calculate the minimum number of animals necessary in each group to provide statistically detectable differences based on antioxidant levels obtained in a pilot study. Animals were procedure naïve and weighed an average of 0.55 kg +/− 0.02 at the initiation of the study. Paired monkeys were housed in stainless steel cages with wire mesh bottoms within the CNPRC exposure chamber facility under high efficiency particulate air (HEPA) filtered conditions starting at 1–2 days following birth. Feeding and enrichment were provided according to the CNPRC standard operating procedure for nursery-reared infants including hand-feedings of human infant formula every 2 h until 5 weeks of age, when hanging bottles were provided. Water was available *ad libitum* and solid foods including monkey chow and fruit were provided twice per day starting at 2 weeks of age. For enrichment, a variety of swings, hanging perches and age-appropriate toys were offered. Temperature and humidity ranges were controlled along with 12 h light and dark cycles.

**Figure 1:**
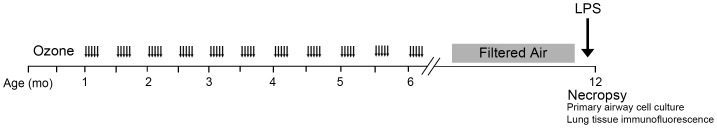
Experimental timeline for postnatal episodic ozone exposure and *in vivo* LPS challenge in juvenile monkeys. Starting at 1 month of age, infant rhesus monkeys (n = 8) were exposed to 11 cycles of ozone. Each cycle consisted of ozone exposure for 5 days (0.5 parts per million at 8 h/day for 6 months (small arrows)) followed by 9 days of filtered air. At the end of 11 exposure cycles, animals were housed in filtered air until 1 year of age. Control animals (n = 9) were housed in filtered air until 1 year of age. An aerosolized LPS challenge via face mask was given to the ozone-exposed and the filtered air-control animals at 1 year of age (n = 4 each group; marked by the large arrow in figure). All animals were necropsied within 24 h of *in vivo* LPS; lung tissues were collected for cell culture and histology.

Details regarding the ozone exposure regimen for animals used in this study have been previously described [20]. In brief, animals (n = 8) received 11 successive cycles of ozone exposure from 1 month to 6 months of age (Figure 1). Animals were treated as an entire group within CNPRC exposure chambers. Each cycle consisted of 5 days of ozone (0.5 parts per million at 8 h/day) followed by 9 days of filtered air. Ozone was generated as previously described [20], [29], [30]. Oximetry and heart rate were monitored by CNPRC veterinary staff during exposure procedures, with no adverse effects reported. At completion of the ozone exposure regimen, all animals remained in filtered air housing for a period of 6 months. Control animals were nursery-raised indoors under HEPA filtered conditions until 1 year of age (n = 9). CNPRC nursery and inhalation and exposure staff monitored the well-being of all study animals with frequent health observations each day including assessment of appetite and body weight. All care is under the direct guidance of CNPRC veterinarians who set the criteria for modifying and/or terminating procedures to ameliorate suffering.

At 1 year of age, a subset of ozone or filtered air control juvenile animals received a single *in vivo* LPS aerosol challenge (filtered air n = 4, ozone n = 4). A dose of 25,000 endotoxin units in PBS (*E. coli* O26:B6; Sigma- Aldrich, St. Louis, MO) was administered via face mask in the morning ∼24 h prior to necropsy. The same commercial lot of LPS was used for all animals and cultures in this study. For sample collection prior to necropsy, animals were anesthetized with an intramuscular dose of ketamine (10 mg/kg); the dose was adjusted as deemed necessary by attending the veterinarian. Animals were euthanized by intravenous overdose of sodium pentobarbitol IV (60 mg/kg). Lung tissue specimens collected at necropsy were immediately processed for primary cell culture and histology (see subsequent sections). These experiments were conducted over a 3 year period with filtered air controls completed in years 1 and 2, ozone exposure groups in year 2 and LPS groups in year 3.

### Primary Airway Epithelial Cell Culture

Airway epithelial cells were isolated from trachea and large bronchi collected at necropsy using protease digestion as described in [30]. Isolated epithelial cells were plated at a density of 4×10^5^ cells on 6.5 mm, 0.4 µm pore size transwell clear polyester membrane inserts (Corning, Corning, NY) coated with FNC coating mix (Athena Enzyme Systems, Baltimore, MD). Cells were first expanded in growth media (BEGM, Lonza, Walkersville, MD) ed with retinoic acid (50 nM Sigma-Aldrich Corp., St. Louis, MO) and upon confluency (approximately 2 weeks), cultured under air-liquid interface conditions for an additional seven days to achieve differentiation according to previously established methods [31]. Polarization of cultures was confirmed by measurement of transepithelial resistance using an STX2 Electrode (World Precision Instruments Inc., Sarasota, FL). Prior to *in vitro* LPS treatment, cultures underwent a complete media change, followed by addition of LPS in media supplemented with 0.5% fetal bovine serum (Invitrogen, Carlsbad, CA) on the apical surface (100 µL/well). After a one hour incubation with LPS, cultures were subsequently washed and incubated for 6 or 24 h with fresh culture media added to the apical surface. We have previously shown that this dose and LPS treatment protocol activates the toll-like receptor signaling pathway in airway epithelial cells as early as 3 h post-treatment and triggers IL-6 as well as IL-8 responses [30].

### RNA Isolation and Analysis

RNA and microRNA were isolated from primary airway epithelial cell cultures using Trizol reagent (Invitrogen). cDNA was generated from total RNA using random hexamer primers and MultiScribe Reverse Transcriptase (Applied Biosystems, Foster City, CA). IL-6, IL-8, TLR4 and GAPDH mRNA was measured by Taqman Real-time PCR methods, using rhesus-specific primer-probe assays that map with 100% identity to the human homologue (Applied Biosystems) and detected using an Applied Biosystems PRISM 7900 Sequence Detection System. Progressive dilution of corresponding purified human cDNA plasmid constructs (Origene, Rockville, MD) for each gene was used to generate standard curves and allow absolute quantitation of copy number. Gene copy numbers are reported relative to GAPDH values.

Putative microRNAs (miR) that target the IL-6 mRNA 3′UTR were identified by the TargetScan 5.1 program which is based on previously-defined algorithms [32], [33]. miR-149, miR-202 and miR-410 were selected based on conservation within the mammalian species and a low total context score (<−0.1) for human IL-6 3′ UTR (Supporting Data Figure 4). miR-let-7a was also included in the analysis as it is a known functional modulator of IL-6 expression [34]. The Taqman MicroRNA Reverse Transcription Kit was used for reverse transcription of hsa-miR-let7a, hsa-miR-149-5p, hsa-miR-202-3p and hsa-miR-410 with target-specific stem loop structure primers (Applied Biosystems). The small nuclear RNA, RNU-6b was amplified as an endogenous control. Analyses using specific microRNA probe sets and amplification reagents were performed on an ABI 7900 (Applied Biosystems). Results for miR data analyses were calculated by use of the deltaCT method, where deltaCT values =  CT value of target miR - CT value of endogenous control RNU-6b.

### Immunofluorescence Staining

Trachea specimens from necropsy were embedded in O.C.T. compound (Sakura Finetek, Torrance, CA) and immunostained as previously described [30] with the following modifications: sections were fixed in acetone, blocked in bovine serum albumin (Vector Laboratories, Burlingame, CA) and stained with a FITC-conjugated mouse anti-human IL-6 (clone MQ2-13A5; eBiosciences, San Diego, CA). A FITC-conjugated mouse IgG1 was used as an isotype control (eBiosciences). Both control and IL-6 antibodies were used at a concentration of 10 µg/ml with an overnight incubation.

### Analysis of microRNA Function

The immortalized human bronchial airway epithelial cell line BEAS-2B S.6 [35] was transfected with 30 nM *mir*Vana mimics of hsa-miR-149-5p, hsa-miR-202-3p, hsa-miR-410 and the miR#1 negative control (lacking any mammalian target) using lipofectamine (Applied Biosystems). After 24 h, transfected cells were evaluated for microRNAs, IL-6 mRNA and IL-6 protein levels. For microRNA binding analysis, the papilloma virus–immortalized human bronchial epithelial cell line HBE [36] was transfected with a 3′ UTR IL-6 gene target cloned upstream of a Firefly luciferase reporter in a dual luciferase reporter system (GeneCopoeia, Rockville, MD) using lipofectamine. HBE cells were co-transfected with the IL-6 3′ UTR clone and 40 nM microRNA mimics, followed by evaluation of relative luciferase activity after 24 h. For transfection-normalization across samples, the firefly luciferase is reported relative to an internal control, consisting of renilla luciferase driven by a CMV promoter.

### Cytokine ELISA

IL-6 and IL-8 protein concentration in apical supernatants collected from primary airway epithelial cell cultures were measured by ELISA Ready-SET-Go! Kits (eBioscience, San Diego, CA). The limit of detection for ELISA assays was 2 pg/mL (IL-6), and 4 pg/mL (IL-8).

### Statistics

All data are reported as mean +/− standard error (SE). Treatment and exposure differences were evaluated on log-transformed values using two-way ANOVA or Student's t-test when appropriate with GraphPad Prism software (GraphPad 5.0, La Jolla, CA). A *p* value of 0.05 or less was considered statistically significant.

## Results

### The Cytokine Response to LPS in Juvenile Airway Epithelial Cells is Altered by Postnatal Episodic Ozone Exposure

To determine if chronic ozone exposure during postnatal development results in persistent changes to innate immune function of airway epithelium, we established primary airway epithelial cell cultures from juvenile rhesus monkeys exposed as infants to episodic ozone. In addition to the effects of ozone, a subset of animals was also challenged *in vivo* with a single dose of aerosolized LPS (Figure 1). Primary cultures were evaluated for cytokine synthesis following *in vitro* treatment with LPS, focusing on IL-6 and IL-8 as measures of prototypic proinflammatory responses. A limited portion of the results from the filtered air control and filtered air + *in vivo* LPS animal groups has been previously reported as part of a manuscript examining the role of chronologic age on epithelial cell innate immune function [30]; the data are included here for exposure-related comparisons.

**Figure 2:**
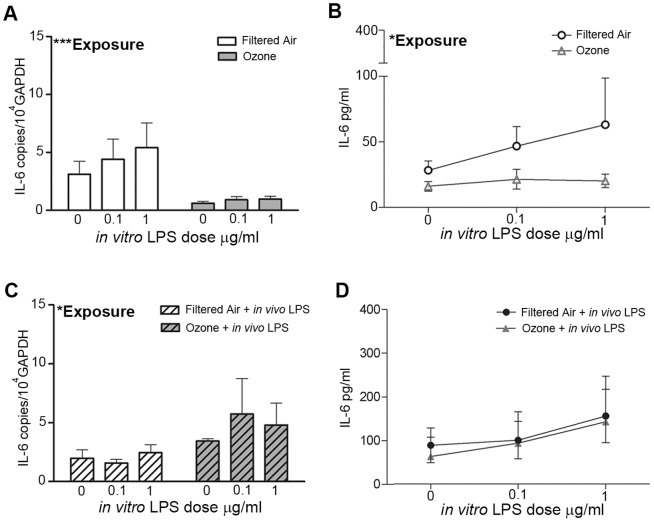
Effect of postnatal ozone exposure on IL-6 expression in juvenile monkey airway epithelial cell cultures following LPS treatment. Airway epithelial cells harvested from one-year-old juvenile rhesus monkeys with prior ozone and/or LPS exposure, as described in Figure 1 were cultured under air-liquid interface conditions and subsequently treated with increasing doses of LPS *in vitro*. Cultures were evaluated for IL-6 mRNA (A, C) and protein (B, D) expression at 24 h post-treatment. Results show the average +/− SE. *p<0.05, *** p<0.001 by two-way ANOVA comparing *in vivo* exposure and *in vitro* LPS concentration (n = 4 for each group except filtered air controls n = 5).

Airway epithelial cells cultured from animals with a history of postnatal ozone exposure exhibited altered synthesis of IL-6 in response to *in vitro* LPS treatment. Significantly reduced IL-6 mRNA (*p* = 0.0002) and protein (*p* = 0.02) levels were observed 24 h post- *in vitro* LPS treatment in airway epithelial cell cultures with a history of ozone exposure as compared to epithelial cells derived from filtered air control animals (Figure 2A–B; two-way ANOVA for *in vivo* exposure and *in vitro* LPS concentration). In epithelial cells from ozone-exposed animals that received a single *in vivo* LPS challenge (ozone + *in vivo* LPS), IL-6 mRNA levels were significantly greater (*p* = 0.01) than their corresponding filtered air + *in vivo* LPS controls and ozone-only animals (Figure 2C; two-way ANOVA for *in vivo* exposure and *in vitro* LPS concentration). The IL-6 protein response to *in vitro* LPS stimulation was not significantly different in the exposure groups that received the *in vivo* LPS challenge (Figure 2D), but there was a trend towards increased IL-6 protein relative to the ozone alone group (p = 0.055 by two-way ANOVA for *in vivo* exposure and *in vitro* LPS concentration). Significant exposure-dependent differences were also observed in the IL-6 mRNA, but not protein response at 6 h post- *in vitro* LPS treatment (Figure S1; *p = 0.01* by two-way ANOVA for *in vivo* exposure and *in vitro* LPS concentration). Although exposure did not significantly affect IL-6 protein levels at the 6 h time point, *in vitro* LPS concentration was a significant source of variation in filtered air and ozone cultures (Figure S1B; *p = 0.04*).

**Figure 3:**
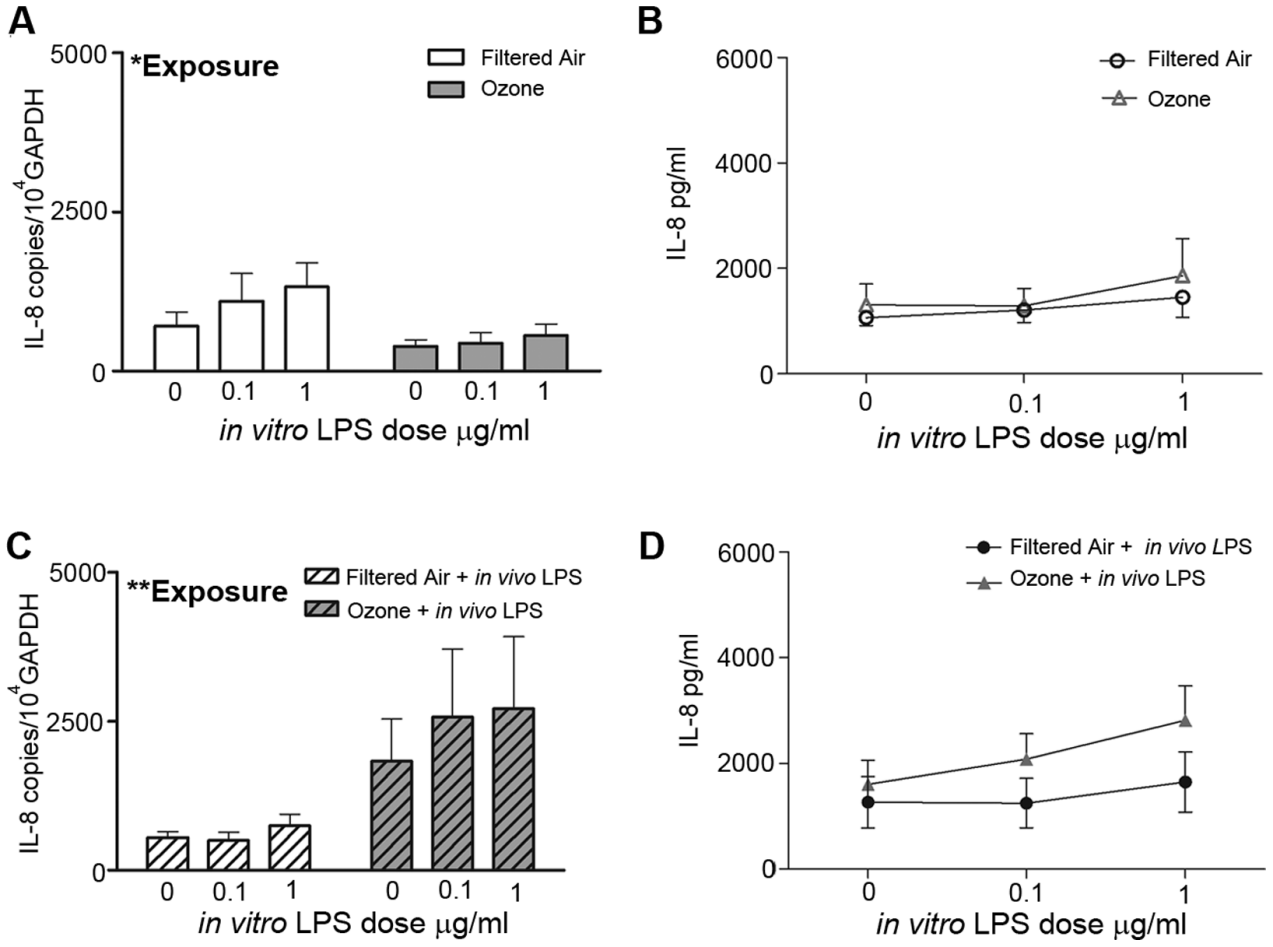
Effect of postnatal ozone exposure on IL-8 expression in juvenile monkey airway epithelial cell cultures following LPS treatment. Airway epithelial cells harvested from one-year-old juvenile rhesus monkeys with prior ozone and/or LPS exposure, as described in Figure 1 were cultured under air-liquid interface conditions and subsequently treated with increasing doses of LPS *in vitro*. Cultures were evaluated for IL-8 mRNA (A, C) and protein (B, D) expression at 24 h post-treatment. Results show the average +/− SE. *p<0.05, **p<0.01 by two-way ANOVA comparing *in vivo* exposure and *in vitro* LPS concentration (n = 4 for each group except filtered air controls n = 5).

The IL-8 response to *in vitro* LPS treatment was similar to IL-6, in that the epithelial cells from the ozone-only exposed animals exhibited attenuated IL-8 mRNA synthesis (*p* = 0.02) and cultures established from ozone + *in vivo* LPS animals showed the greatest IL-8 mRNA induction (*p* = 0.002) (Figure 3A, C; two-way ANOVA for *in vivo* exposure and *in vitro* LPS concentration). The IL-8 protein response was not significantly affected by exposure, although there was a trend towards increased IL-8 secretion in the ozone + *in vivo* LPS group relative to filtered air + *in vivo* LPS (Figure 3 B, D, p = 0.085 two-way ANOVA for *in vivo* exposure and *in vitro* LPS concentration). Similar exposure-dependent differences were observed in the IL-8 mRNA but not protein response at 6 h post- *in vitro* LPS treatment (Figure S2).

**Figure 4:**
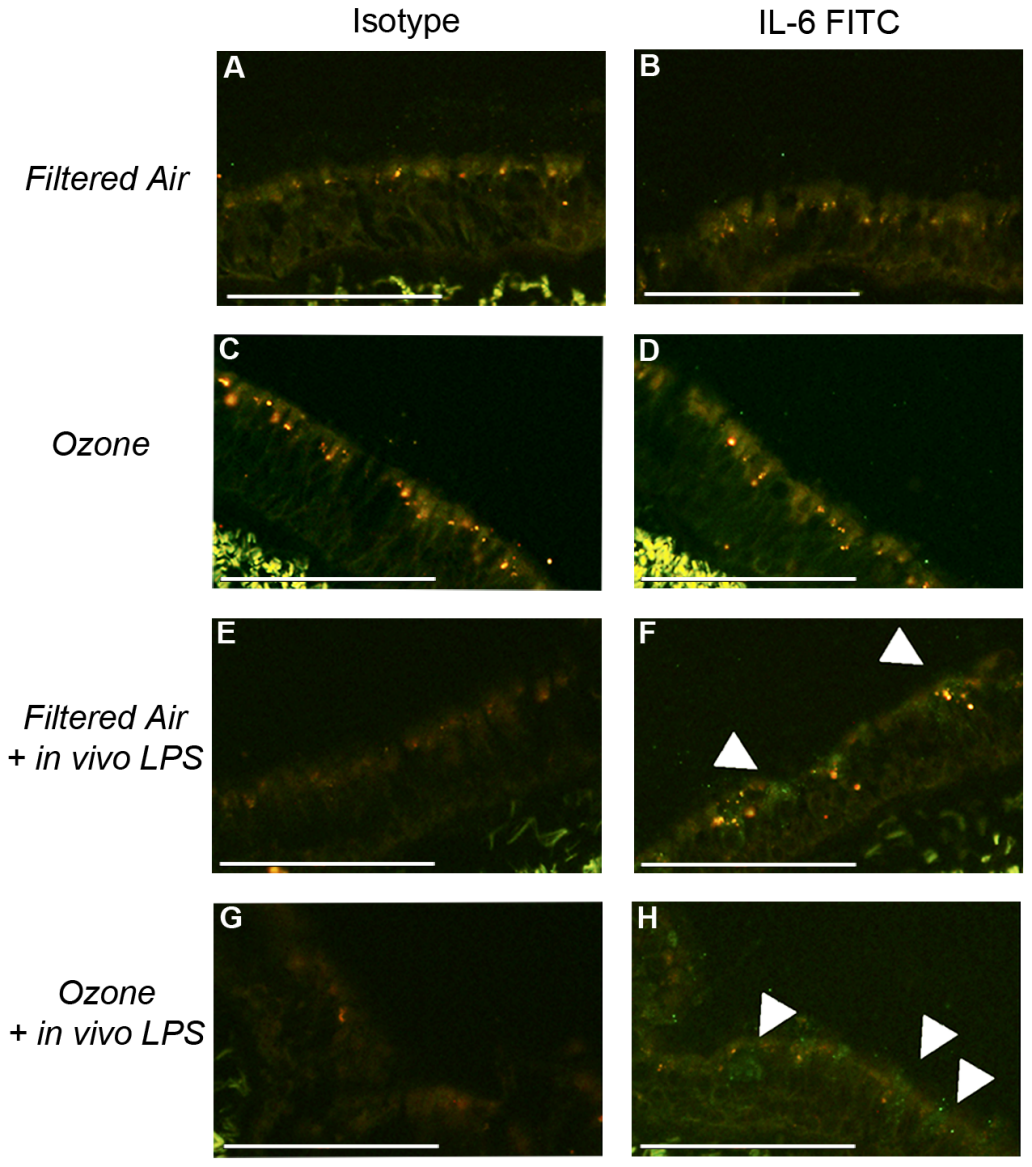
IL-6 immunofluorescence staining in juvenile monkey trachea following postnatal ozone and/or *in vivo* LPS. Trachea cryosections from a representative animal in each exposure group were stained with FITC-conjugated anti-human IL-6 mouse monoclonal antibody (B, D, F, and H). FITC-conjugated mouse IgG1 isotype controls are included for comparison (A, C, E, and G). Images were collected at 20× magnification. Scale bar =  100 µm. White arrows indicate IL-6 staining.

To determine if cytokine expression measured in primary airway epithelial cell cultures was reflective of airway epithelium *in vivo*, we conducted immunostaining of trachea cryosections obtained from the same animals used as the source of primary cell cultures (Figure 4). Focusing on IL-6, we detected positive immunofluorescence in animals that received *in vivo* LPS, including filtered air controls and those with a history of postnatal ozone exposure (Figure 4F, H). Little to no IL-6 staining was observed in animals that did not undergo *in vivo* LPS challenge (Figure 4B, D).

TLR4 recognizes LPS and mediates the subsequent cytokine response [37]. TLR4 has also been identified as an essential susceptibility gene for inflammatory and physiologic effects of ozone exposure in certain mouse strains [38], [39], [40]. To determine if our findings relative to postnatal ozone exposure were associated with altered TLR4 expression in airway epithelium, we measured TLR4 copy number in primary cultures and found no significant exposure-related differences (Figure S3).

### Effect of Postnatal Ozone on microRNA Targets for IL-6 3′ UTR

MicroRNAs are small (21–23 nucleotides) noncoding RNAs that regulate gene expression by affecting mRNA stability and protein synthesis through post-transcriptional modifications [41]. Recent expression profiling studies show that environmental pollutants can change the expression of microRNAs in the lung [42], [43]. Given the discordant IL-6 mRNA and protein responses observed in airway epithelial cell cultures established from animals with a history of postnatal ozone and/or LPS exposure, we sought to determine if microRNAs that putatively target IL-6 were differentially expressed in association with antecedent ozone or LPS. miR-149, miR-202, and miR-410 were identified using TargetScan 5.1 (Figure S4), and miR-let7a expression was also measured as it is a known functional modulator of IL-6 expression [34].

**Figure 5:**
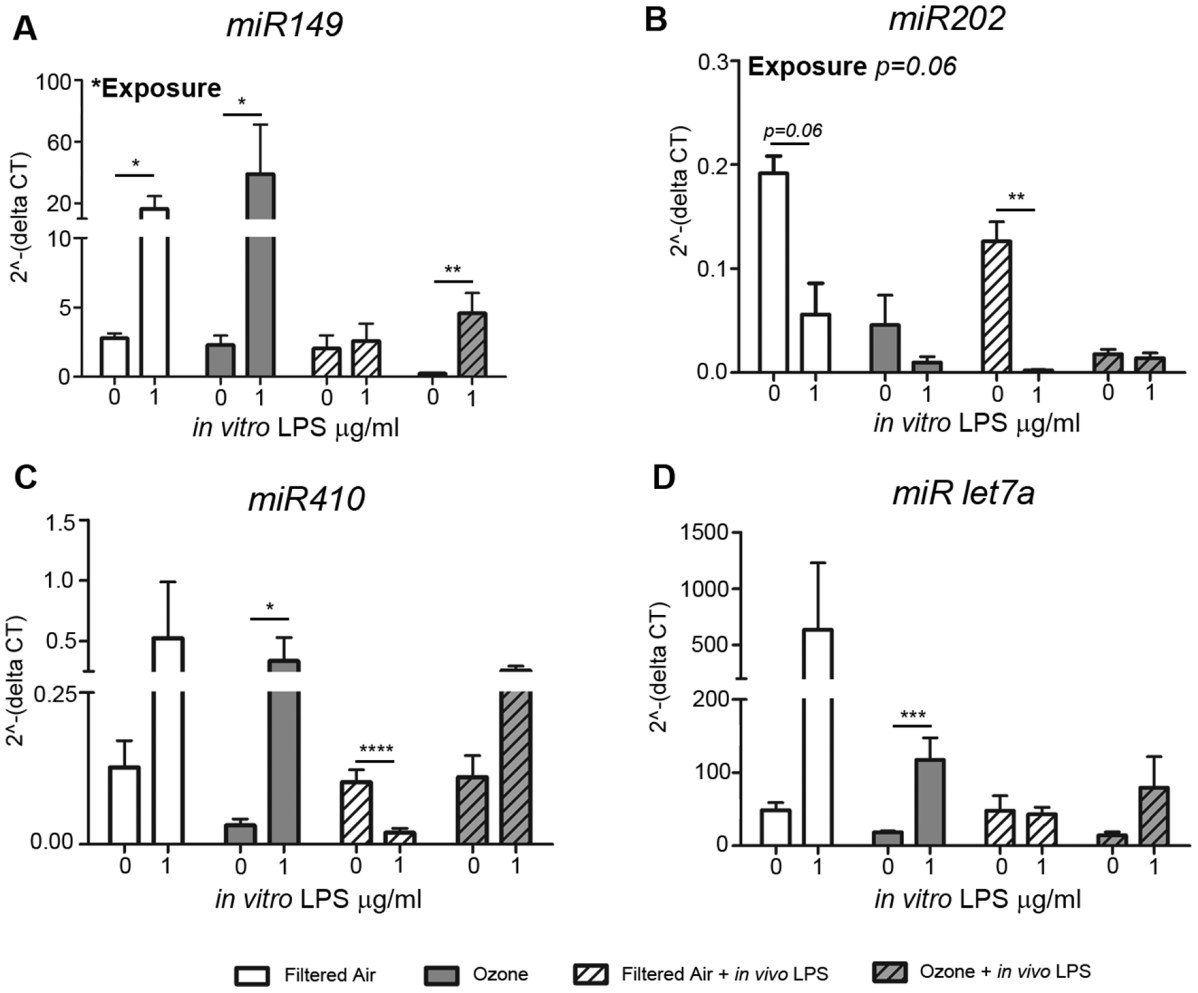
Effect of postnatal ozone exposure on miR-149, miR-202, miR-410 and miR-let7a expression in juvenile monkey airway epithelial cell cultures. Comparison of constitutive (0 µg/ml) and LPS-induced (1 µg/ml, 6 h post-treatment) microRNA expression in primary airway epithelial cell cultures derived from one-year-old juvenile rhesus monkeys with prior ozone and/or LPS exposure as described in Figure 1. (A) miRNA-149, (B) miRNA-202, (C) miRNA-410, (D) miRNA-let7a. Graphs show the average +/− SE of the 2?-(delta CT) where the delta CT =  (microRNA of interest – endogenous control microRNA (RNU-6b)). microRNAs were extracted from n = 3–5 animals per group. Results from Student's t-tests comparing microRNA expression at baseline and post- *in vitro* LPS treatment are shown with horizontal bars. *p<0.05, **p<0.005, ***p<0.0003.

Most of the evaluated microRNAs showed significant ozone/*in vivo* LPS exposure- or *in vitro* LPS treatment-dependent expression differences in primary airway epithelial cell cultures (Figure 5). In untreated cultures (0 µg/ml), miR-149 showed the most significant difference in expression levels based upon prior exposure history, with epithelial cells from ozone + *in vivo* LPS animals having significantly reduced miR-149 as compared to all other exposure groups (Figure 5A, *p* = 0.02; two-way ANOVA for *in vivo* exposure and *in vitro* LPS treatment). *In vitro* LPS treatment (1 µg/ml) was also a significant source of variation for miR-149 expression (p<0.0001). At 6 h post-LPS treatment *in vitro,* miR-149 expression significantly increased above baseline in epithelial cells from all exposure groups except filtered air controls that received an *in vivo* LPS challenge. Similar to miR-149, miR-202 expression at baseline was lowest in the ozone + *in vivo* LPS group, with exposure-dependent effects that were near statistical significance (Figure 5B; two-way ANOVA for *in vivo* exposure and *in vitro* LPS treatment; *p* = 0.06 for exposure and *p* = 0.04 for LPS). miR-202 decreased with *in vitro* LPS treatment compared to baseline values except in cultures with a history of ozone exposure (*p* = 0.06 for ozone alone, *p* = 0.002 for ozone + *in vivo* LPS). For miR-410, epithelial cell cultures derived from ozone-only animals showed the lowest constitutive expression levels but these were significantly increased with *in vitro* LPS treatment (Figure 5C; *p* = 0.05). In contrast, *in vitro* LPS treatment significantly reduced miR-410 expression in animals that were exposed to LPS *in vivo* (p<0.0001). *In vitro* LPS treatment but not prior *in vivo* exposures was a significant source of variation for miR-let-7a expression with significantly increased miR-let-7a levels in ozone-exposed animals following LPS treatment *in vitro* (Figure 5D; *p* = 0.001; two-way ANOVA for *in vivo* exposure and *in vitro* LPS treatment). Collectively, our data indicate that postnatal episodic ozone alters microRNA expression profiles in juvenile airway epithelial cells, both constitutively as well as in response to *in vitro* LPS treatment. Further, antecedent LPS (from *in vivo* challenge) also affects the microRNA response to secondary LPS treatment in airway epithelial cells.

### Binding of microRNA Targets to the IL-6 3′ UTR and microRNA Modulation of IL-6 Expression

**Figure 6:**
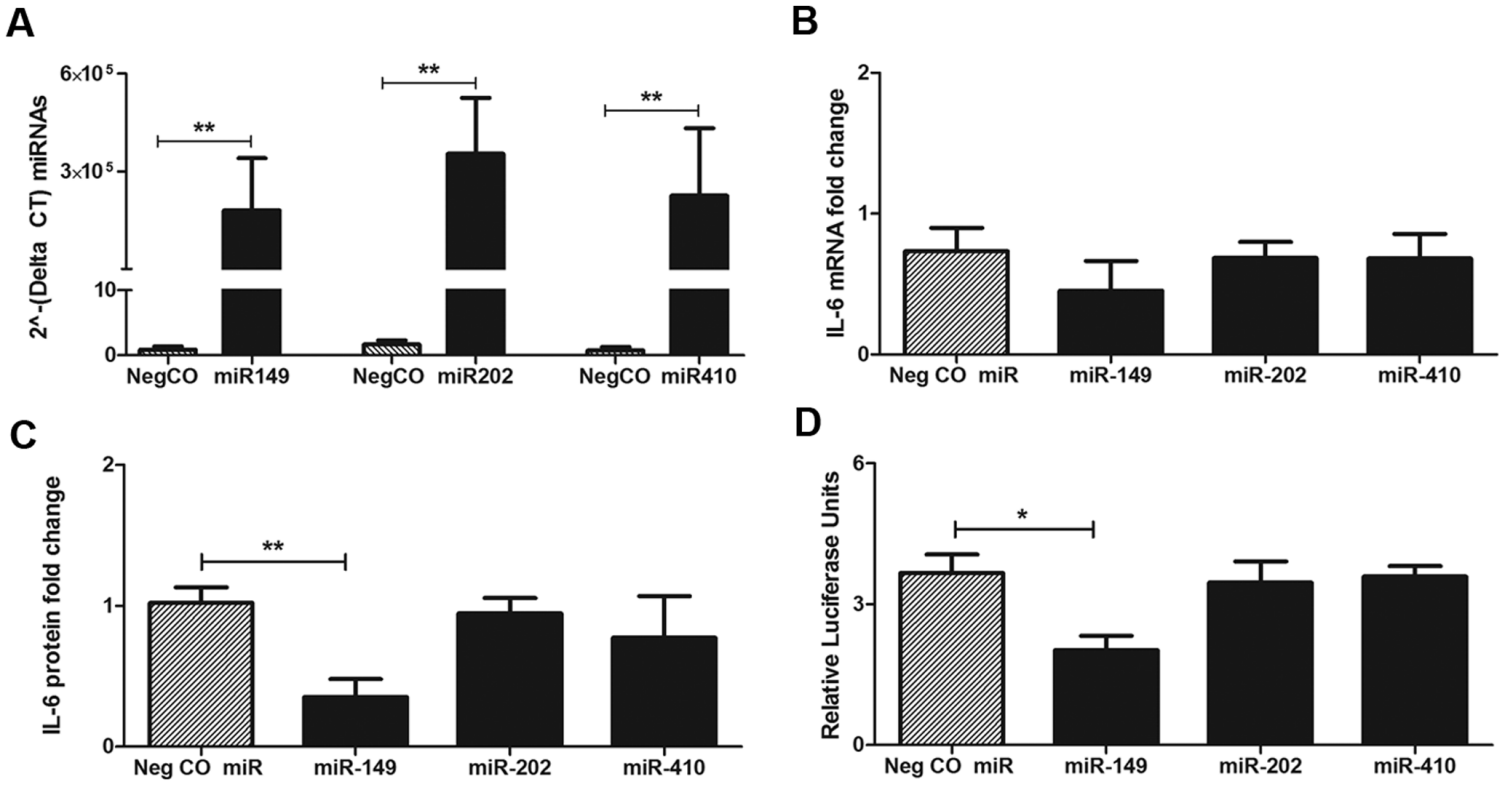
Assessment of microRNA regulation of IL-6 in human airway epithelial cell cultures. To evaluate the ability of the putative IL-6-targeting microRNAs to regulate expression of IL-6, the BEAS-2B cell line was transfected with a negative control (NegCO) microRNA or mimics for miR-149, miR-202 or miR-410. (A) Expression of microRNAs in BEAS-2B 24 h post-transfection with a NegCO microRNA or mimics for miR-149, miR-202, or miR-410. Values are reported as the average +/− SE of the 2?-(delta CT) relative to the endogenous control microRNA RNU-6b. (B) IL-6 mRNA and (C) IL-6 protein expression was evaluated in transfected BEAS-2B cells after 24 h. Data are reported as the fold-change in IL-6 mRNA or IL-6 protein as compared to no mimic controls for each experiment. (D) Binding of microRNA mimics to IL-6 mRNA was tested in the HBE1 cell line with the IL-6 3′UTR cloned into a firefly/renilla luciferase plasmid reporter system. For transfection normalization across samples, the relative luciferase units are reported for firefly versus renilla luciferase. Data are from 4 separate experiments. *p<0.05, **p<0.005 by Student's t-test.

To evaluate the ability of putative IL-6 targeting microRNAs (miR-149, miR-202, miR-410) to modulate IL-6 mRNA and/or protein expression, the human bronchial epithelial cell line, BEAS-2B S.6 was transfected with microRNA mimics (Figure 6A). BEAS-2B S.6 were chosen for these functional experiments due to their ability to synthesize IL-6 both constitutively and in response to LPS treatment. At 24 h post-transfection, cells transfected with miR-149 showed minimal changes to IL-6 mRNA levels but had secreted significantly less IL-6 protein as compared to cells transfected with the negative microRNA control (Figure 6B–C, *p* = 0.004). Comparatively, transfection with miR-202 or miR-410 did not significantly affect IL-6 mRNA or protein levels. To assess specificity of microRNA binding, the human bronchial epithelial cell line HBE (which expresses little to no endogenous IL-6) was co-transfected with microRNA mimics and an IL-6 gene target luciferase reporter plasmid (Figure 6D). Of the microRNA mimics tested, only miR-149 showed significant reduction of relative luciferase units (*p* = 0.01), indicating the ability of this microRNA to bind to the IL-6 3′ UTR. Relative luciferase units observed in HBE cells co-transfected with IL-6 gene target luciferase reporter plasmid and miR-202 or miR-410 were similar to transfection with the negative microRNA mimic.

## Discussion

There is a very limited understanding of the mechanisms by which environmental perturbations during infancy affect pulmonary immunity later in adulthood, particularly in primate species. To address this deficiency, we investigated the immunomodulatory impact of early life air pollutant exposure using the rhesus monkey as an animal model of childhood development. We have previously shown that episodic exposure of infant monkeys to ozone followed by longitudinal evaluation as juveniles (at one year of age) resulted in attenuation of *in vivo* airway responses to LPS challenge, despite housing for 6 months exclusively in filtered air [20]. Furthermore, *in vitro* LPS treatment of peripheral blood mononuclear cells from juvenile animals with a history of postnatal ozone resulted in attenuation of both IL-6 and IL-8 protein synthesis, suggesting that air pollutant exposures may dampen systemic immune responses in an epigenetic fashion. In this current study, we have focused on the airway epithelium as an additional putative target for persistent immunomodulation by ozone. Using *in vitro* cultures of primary airway epithelial cells isolated from juvenile monkeys with a history of ozone exposure during postnatal development, we observed dysregulation of cytokine responses to *in vitr*o LPS treatment and alterations in microRNA expression in association with exposure history.

Our finding of attenuated inflammatory responses to bacterial LPS in airway epithelium of rhesus monkeys previously exposed to ozone is consistent with published effects of ozone reducing host defense mechanisms during microbial infection of murine models [7], [10], [11]. However, it is important to emphasize that the animals evaluated in this study were housed under HEPA-filtered conditions for a period of six months after ozone exposure, indicating that the observed effects were not the result of recent exacerbation; rather, the functional change in epithelial cells from ozone-exposed infant airways was retained with increasing chronological age. We believe that our study is the first to report a persistently immunomodulated airway epithelial cell phenotype in response to a defined experimental exposure during postnatal development. The observed differential IL-6 and IL-8 mRNA expression in association with ozone highlights the inherent alterations that exposures have on epithelial cell immune function. Early life ozone exposure appears to reprogram the airway epithelium such that it synthesizes less IL-6 and IL-8 mRNA as compared with filtered air control counterparts following treatment with LPS (Figure 2A, 3A). Interestingly, only IL-6 protein was significantly modulated by prior ozone exposure, whereas IL-8 protein secretion in response to LPS was not consistently affected (Figures 2B, 3B). The role of discordant mRNA and protein expression in airway epithelium is unclear but may be due to a delay in IL-8 protein translation relative to IL-6 that was not captured in our experimental time frame. Although our current study was limited to IL-6 and IL-8, we postulate that other proinflammatory cytokines, including TNF-α and IL-1β, may also be affected.

In addition to the impact of ozone on airway epithelium, we also observed that prior *in vivo* challenge with a single LPS aerosol significantly affected the cytokine response elicited by primary airway epithelial cell cultures. We have recently reported that chronological age and antecedent LPS exposure can have a significant effect on the ability of airway epithelium to respond to subsequent LPS exposure, resulting in a reduction of IL-6 mRNA but increased secretion of IL-6 protein [30]. Here, *in vivo* aerosol LPS exposure at one year of age in animals with prior ozone history elicited significantly higher IL-6 and IL-8 mRNA levels in primary airway epithelial cells constitutively and upon secondary LPS treatment *in vitro*, as compared with ozone alone counterparts (Figures 2C,3C). This finding indicates that the airway epithelial cell cytokine response is affected by both the type and sequence of exposures. Cytokine responses in our *in vitro* LPS epithelial cell cultures may reflect the timing and dose of the *in vivo* LPS challenge, as tracheal tissues were harvested from all animals approximately 24 h after aerosolized LPS exposure, and our previous work shows that significant pulmonary inflammation lasts for at least 24 h after a single LPS dose is administered [20]. The observation that IL-6 protein was detected exclusively in trachea cryosections from animals that received inhaled LPS (Figure 4), suggests that the patterned cytokine response was established *in vivo*. While it is unclear why *in vitro* LPS treatment of airway epithelial cells derived from animals that only have postnatal ozone exposure does not recapitulate the effects of *in vivo LPS*, it may be speculated that interactions with other cells in the lung microenvironment (i.e. monocytes/macrophages) may impart a persistent cytokine phenotype in epithelium. Indeed, we have previously reported that postnatal ozone exposure resulted in elevated monocyte frequencies in bronchoalveolar lavage that persisted with maturity [20]. Taken together, our data emphasize the importance of studying the effects of co-exposures during this early life period given that environmental exposures likely involve multiple components which may act synergistically at a molecular level and result in distinct immunomodulation.

Accumulating evidence suggests that epigenetic mechanisms may play a role in persistent modification of host responses following air pollution exposure [28]. Recently, microRNAs have been identified as regulators of RNA responses in various biological processes including inflammatory signaling via TLRs [44]. In this study we focused our investigation on microRNAs that may regulate IL-6, based upon the identification of microRNAs that directly or indirectly affect IL-6 expression in other cell types [34], [45], [46], [47], [48], [49], [50]. miR-149 expression was significantly dependent upon exposure, as airway epithelial cells derived from animals with a history of postnatal ozone + *in vivo* LPS exhibited reduced constitutive levels relative to other animal groups (Figure 5A). There was also a trend towards exposure-dependent differences in miR-202 expression in airway epithelium, with reduced levels in cultures derived from animals with prior ozone exposure (Figure 5B). Upon *in vitro* LPS treatment, miR-149, miR-410 and miR-let7a levels increased in epithelial cells from animal groups without *in vivo* LPS; *in vivo* LPS resulted in a comparatively attenuated microRNA response. In contrast, miR-202 expression levels were reduced in response to *in vitro* LPS treatment. In our study, differential expression of miR-149 and miR-let7a may contribute to the distinct IL-6 protein responses of airway epithelial cells based upon postnatal exposure history. The low IL-6 protein response in ozone and filtered air groups was associated with high LPS-induced levels of miR-149 and miR-let7a whereas the high IL-6 protein in the *in vivo* LPS-exposed groups corresponded to lower levels of these miRNAs. Induction of miRs -149 and -let7a in LPS-treated airway epithelial cell cultures may also explain why LPS concentration was a significant source of variation in IL-6 protein levels at 6 but not 24 h. Using microRNA mimics and 3′UTR binding assays, we also found that miRNA-149 was capable of regulating IL-6 protein expression in human airway epithelial cell lines, suggesting that this microRNA may be an important modulator of IL-6 protein expression following LPS exposure of airway epithelium. The current study was limited to microRNAs targeting IL-6 but it is likely that other microRNAs are differentially expressed due to early life environmental exposures and future studies will focus on identifying additional targets that may be impacted by ozone exposure.

A single microRNA is capable of controlling hundreds of different gene targets and individual genes are often targeted by many different microRNAs. Under steady-state conditions, microRNAs appear to act as only moderate regulators, however it is important to consider that microRNAs rarely function through a single gene target and it is the combined regulation of many different genes that determines their true functionality [51]. miR-149, which in our study appears to modulate IL-6 protein expression in airway epithelial cells, has also been shown to target several other diverse genes including E2F1 [52], [53], [54]. E2F1 is a transcriptional activator recruited by NF-kB upon TLR activation to control the LPS inducibility of proinflammatory cytokines [55]. In addition to IL-6, E2F1 may have also been modulated by miR-149 in our epithelial cell cultures, contributing to the observed downregulation of this proinflammatory cytokine. Several gene targets involved in innate immune responses have also been described for both miR-410 and miR-202; it has been speculated that these microRNAs play a role in repressing inflammatory reactions [56], [57], [58].

In conclusion, results from this study show that exposure to ozone in early life intrinsically alters the ability of airway epithelial cells to mount a robust proinflammatory IL-6 response to bacterial LPS challenge. Further, the expression of IL-6-targeting microRNAs is persistently affected by environmental exposures and likely contributes to the dysregulated cytokine response profiles. Because epidemiologic studies strongly support a window of immune susceptibility within the first year of life in humans, it will be important to evaluate other environmental insults for their ability to impose epigenetic reprogramming of airway immune responses.

## Acknowledgments

The authors thank Sarah Davis, Paul-Michael Sosa, Sona Santos, Louise Olsen, and Brian Tarkington for technical support during this project. We also thank Candace Burke and Carolyn Black for helpful discussions during the preparation of this manuscript.

## Supporting Information

Figure S1 **Effect of postnatal ozone on IL-6 expression in juvenile monkey airway epithelial cell cultures 6 h post-LPS treatment.** Airway epithelial cells harvested from one-year-old juvenile rhesus monkeys with prior ozone and/or LPS exposure (as described in Figure 1) were cultured under air-liquid interface conditions and subsequently treated with increasing doses of LPS *in vitro*. IL-6 mRNA and protein expression was evaluated at 6 h post-treatment in filtered air and ozone cultures (A, B) as well as in filtered air + *in vivo* LPS and ozone + *in vivo* LPS cultures (C, D). Results show the average +/− SE. *p<0.05, **p<0.01, *** p<0.001 by two-way ANOVA comparing *in vivo* exposure and *in vitro* LPS concentration (n = 4–5 for each group).
(TIF)

Figure S2 **Effect of postnatal ozone exposure on IL-8 expression in juvenile monkey airway epithelial cell cultures 6 h post-LPS treatment.** Airway epithelial cells harvested from one-year-old, juvenile rhesus monkeys with prior ozone and/or LPS exposure (as described in Figure 1) were cultured under air-liquid interface conditions and subsequently treated with increasing doses of LPS *in vitro*. IL-8 mRNA and protein expression was evaluated at 6 h post-treatment in filtered air and ozone cultures (A, B) as well as in filtered air + LPS and ozone + *in vivo* LPS cultures (C, D). Results show the average +/− SE. *p<0.05, **p<0.01 by two-way ANOVA comparing *in vivo* exposure and *in vitro* LPS concentration (n = 4–5 for each group).
(TIF)

Figure S3 **Comparison of TLR4 expression in juvenile airway epithelial cells from different exposure groups.** Constitutive TLR4 mRNA expression in primary airway epithelial cell cultures derived from juvenile rhesus monkeys exposed postnatally to filtered air, ozone, filtered air + *in vivo* LPS, or ozone + *in vivo* LPS. TLR4 copy number relative to GAPDH was determined by RT-PCR and calculated based on standard curves with the average +/−SE graphed for n = 3–5 per group. 1-way ANOVA for exposure-dependent differences showed no significance.
(TIF)

Figure S4 **Identification of IL-6 targeting microRNAs.** (A) A schematic of human IL-6 mRNA 3′UTR showing three potential binding sites for miR-149, miRlet-7a/miR-202 (overlap), and miR-410. Binding sites are indicated in gray by their position on the 3′UTR. (B) Context scores for rhesus and human microRNAs evaluated in this study are listed as determined by the TargetScan 5.1 program. The context score for each site is the sum of the (1) site-type contribution, (2) 3′ pairing contribution, (3) local AU contribution, and (4) position contribution, as described in Grimson et al. [33]. A lower score indicates more favorable binding.
(TIF)

Checklist S1 (DOC)
