# Supplementary figures and images for: Early Life Ozone Exposure Results in Dysregulated Innate Immune Function and Altered microRNA Expression in Airway Epithelium
Source: PLoS One. 2014 Mar 4;9(3):e90401. doi: 10.1371/journal.pone.0090401 (PMC3942419; doi:10.1371/journal.pone.0090401)

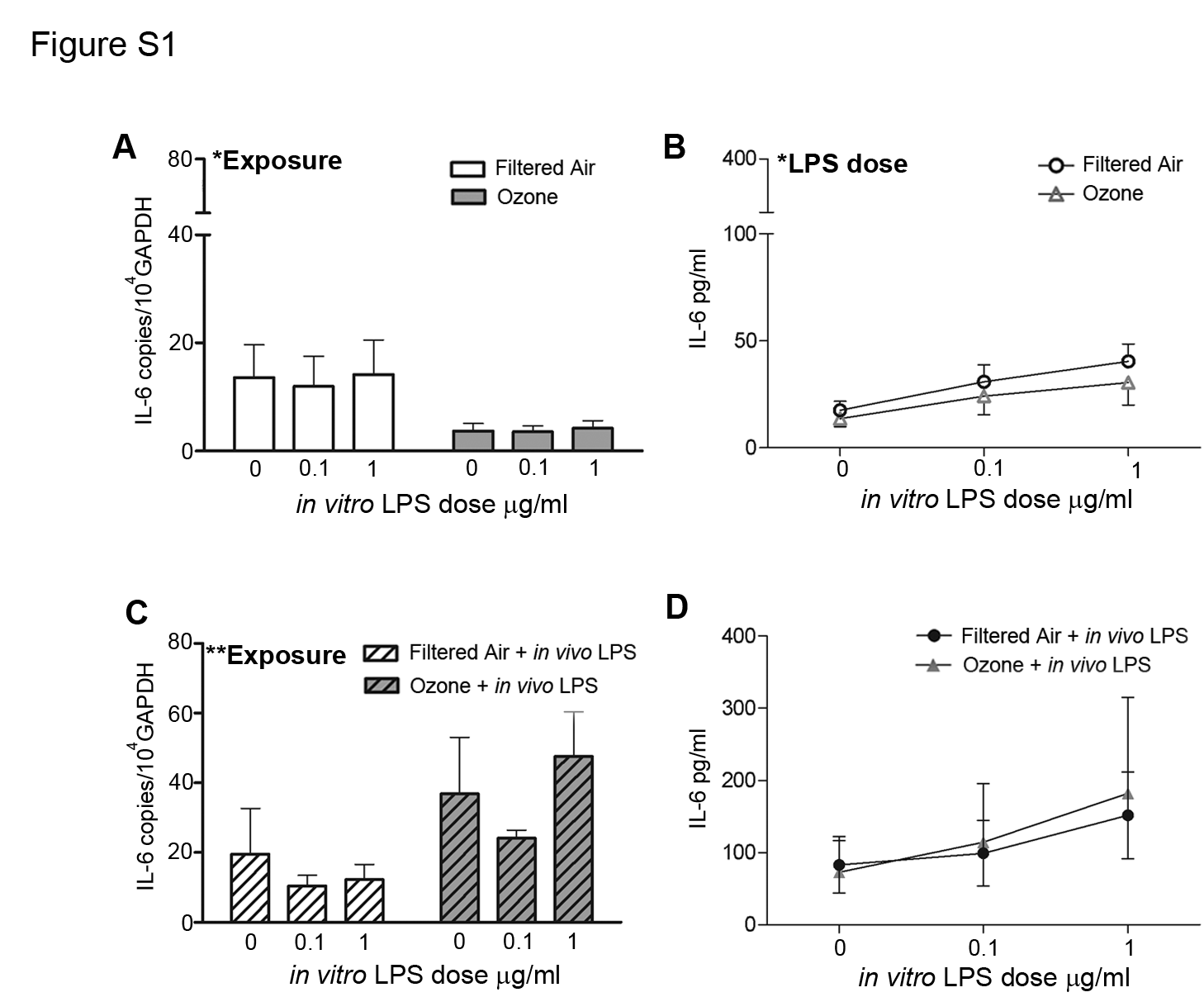

Supplement: Figure S1 — Effect of postnatal ozone on IL-6 expression in juvenile monkey airway epithelial cell cultures 6 h post-LPS treatment. Airway epithelial cells harvested from one-year-old juvenile rhesus monkeys with prior ozone and/or LPS exposure (as described in Figure 1) were cultured under air-liquid interface conditions and subsequently treated with increasing doses of LPS in vitro. IL-6 mRNA and protein expression was evaluated at 6 h post-treatment in filtered air and ozone cultures (A, B) as well as in filtered air + in vivo LPS and ozone + in vivo LPS cultures (C, D). Results show the average +/− SE. *p<0.05, **p<0.01, *** p<0.001 by two-way ANOVA comparing in vivo exposure and in vitro LPS concentration (n = 4–5 for each group). (TIF) [file pone.0090401.s001.tif]

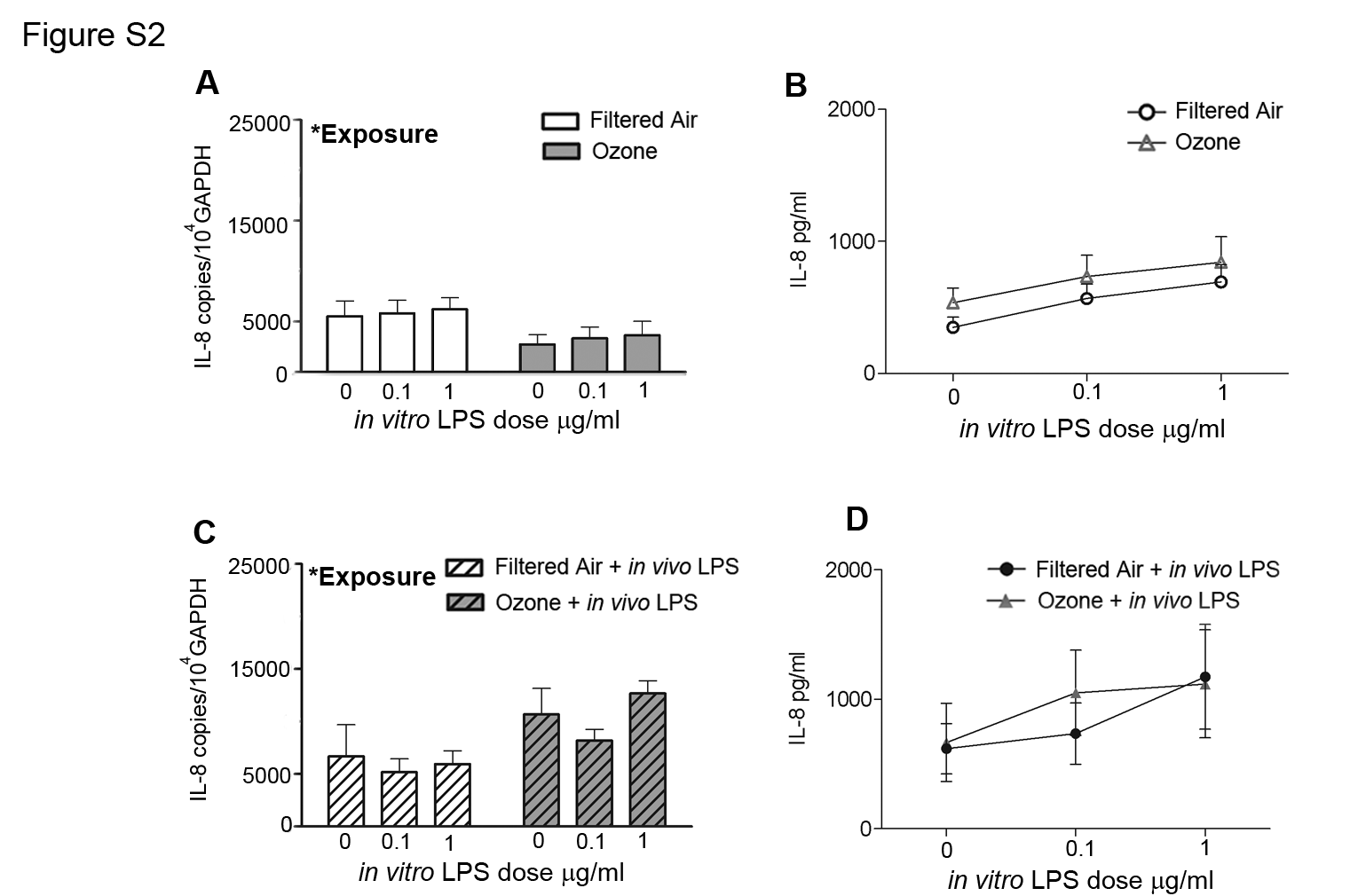

Supplement: Figure S2 — Effect of postnatal ozone exposure on IL-8 expression in juvenile monkey airway epithelial cell cultures 6 h post-LPS treatment. Airway epithelial cells harvested from one-year-old, juvenile rhesus monkeys with prior ozone and/or LPS exposure (as described in Figure 1) were cultured under air-liquid interface conditions and subsequently treated with increasing doses of LPS in vitro. IL-8 mRNA and protein expression was evaluated at 6 h post-treatment in filtered air and ozone cultures (A, B) as well as in filtered air + LPS and ozone + in vivo LPS cultures (C, D). Results show the average +/− SE. *p<0.05, **p<0.01 by two-way ANOVA comparing in vivo exposure and in vitro LPS concentration (n = 4–5 for each group). (TIF) [file pone.0090401.s002.tif]

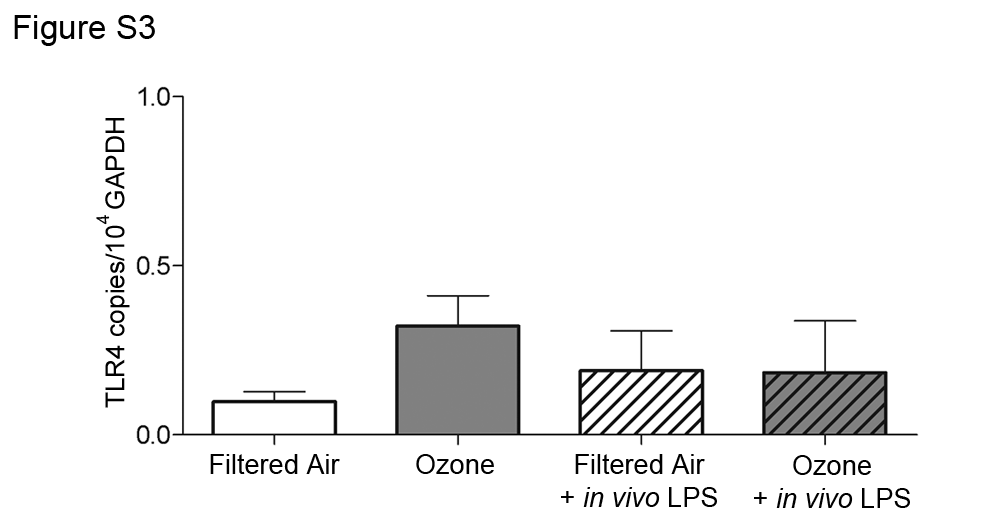

Supplement: Figure S3 — Comparison of TLR4 expression in juvenile airway epithelial cells from different exposure groups. Constitutive TLR4 mRNA expression in primary airway epithelial cell cultures derived from juvenile rhesus monkeys exposed postnatally to filtered air, ozone, filtered air + in vivo LPS, or ozone + in vivo LPS. TLR4 copy number relative to GAPDH was determined by RT-PCR and calculated based on standard curves with the average +/−SE graphed for n = 3–5 per group. 1-way ANOVA for exposure-dependent differences showed no significance. (TIF) [file pone.0090401.s003.tif]

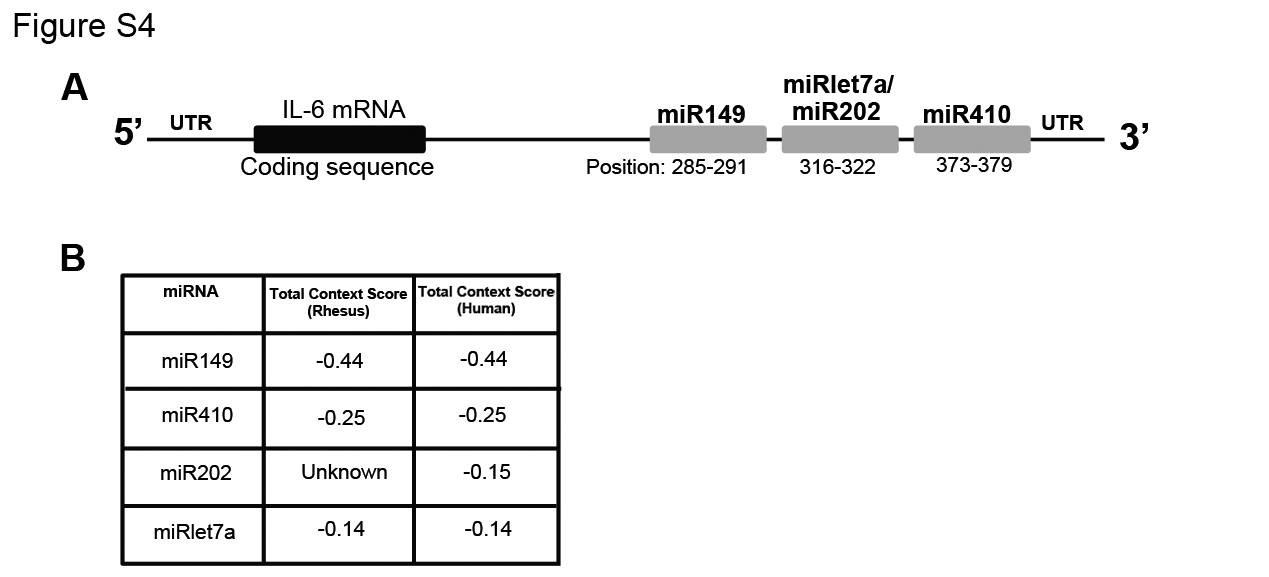

Supplement: Figure S4 — Identification of IL-6 targeting microRNAs. (A) A schematic of human IL-6 mRNA 3′UTR showing three potential binding sites for miR-149, miRlet-7a/miR-202 (overlap), and miR-410. Binding sites are indicated in gray by their position on the 3′UTR. (B) Context scores for rhesus and human microRNAs evaluated in this study are listed as determined by the TargetScan 5.1 program. The context score for each site is the sum of the (1) site-type contribution, (2) 3′ pairing contribution, (3) local AU contribution, and (4) position contribution, as described in Grimson et al. [33]. A lower score indicates more favorable binding. (TIF) [file pone.0090401.s004.tif]
